# Association between neighbourhood poverty and type 2 diabetes risk. Does moving from a high to lower poverty neighbourhood reduce diabetes risk?

**DOI:** 10.23889/ijpds.v9i5.2897

**Published:** 2024-09-18

**Authors:** Sharmin Majumder, Gillian Booth, Rahim Moineddin, Andrew Pinto

**Affiliations:** 1 University of Toronto; 2 ICES (formerly known as the Institute for Clinical Evaluative Sciences); 3 Upstream Lab, MAP/Centre for Urban Health Solutions, Li Ka Shing Knowledge Institute, Unity Health Toronto

## Objectives

Diabetes, a pressing global public health crisis, Diabetes, a global health crisis, is significantly impacted by social determinants, including neighborhood characteristics. This study aims to to assess whether relocation from a high poverty to lower poverty neighbourhood is associated with a reduction in T2D incidence.

## Methods and Results

This population-based, propensity-matched cohort study will use linked administrative health to examine the association between neighborhood relocation and T2D incidence. The study population will include adults (age ≥20 years) residing in high poverty urban neighborhoods between April 1st, 2002, to March 31st, 2021, as defined by an area Low-Income Measure - After Tax value of 38,730 dollars for a household size 4 based on the Canadian Census. Individuals will be followed for a new diagnosis of T2D using a validated algorithm based on hospitalization and physicians’ claims data. Propensity score matching will used to match individuals who moved from high-to-lower poverty neighbourhoods to one of two comparison groups: those moving from high-to-high poverty areas and those who remain in their original neighbourhood. Time-to-event analysis utilizing Cox proportional hazards regression with a robust variance estimator will be used to compare T2D incidence between matched groups. As a sensitivity analysis, non-propensity score modeling will be conducted using neighbourhood of residence as a time-varying covariate. The results of the aforementioned analyses will be presented during the conference.

## Implications

By clarifying the link between neighborhood poverty and T2D incidence, the results will guide focused interventions to alleviate health inequalities in socioeconomically disadvantaged areas.

